# The safety and efficacy of oxaliplatin-loaded drug-eluting beads transarterial chemoembolization for the treatment of unresectable or advanced lung cancer

**DOI:** 10.3389/fphar.2022.1079707

**Published:** 2022-11-28

**Authors:** Yonghua Bi, Fazhong Li, Jianzhuang Ren, Xinwei Han

**Affiliations:** ^1^ Department of Interventional Radiology, The First Affiliated Hospital of Zhengzhou University, Zhengzhou, China; ^2^ Department of Interventional Radiology, Luoyang Central Hospital Affiliated to Zhengzhou University, Luoyang, China

**Keywords:** lung cancer, drug-eluting beads transarterial chemoembolization (DEB-TACE), CalliSpheres beads, oxaliplatin, transarterial chemoembolization (TACE)

## Abstract

**Aim:** Drug-eluting beads are usually applied for the treatment of advanced hepatocellular carcinoma. Oxaliplatin was suggested as first-line therapy for advanced non–small-cell lung cancer. However, there has been little investigation about the application of drug-eluting beads transarterial chemoembolization (DEB-TACE) with oxaliplatin-loaded CalliSpheres beads (CB) for the treatment of unresectable or advanced lung cancer. We aimed to investigate the safety and efficacy of oxaliplatin-loaded DEB-TACE for the treatment of unresectable or advanced lung cancer.

**Methods:** From January 2019 to December 2021, all patients with primary unresectable or advanced lung cancer who underwent DEB-TACE with oxaliplatin-loaded CB were retrospectively enrolled. This study defined overall survival and objective response rate (ORR) as the primary endpoints, disease control rate (DCR) and progression-free survival (PFS) as the secondary endpoints.

**Results:** A total of 33 sessions of DEB-TACE were performed in 20 patients, with a mean of 1.7 ± 1.0 sessions. A total of 55 arteries were emoblized by CB, including 40 bronchial arteries, 13 intercostal arteries, one suprarenal artery and one inferior phrenical artery. No procedural-related mortality or severe complications were observed. The median tumor diameter was 49.0 [Interquartile range (IQR) 37.8–66.8] mm before DEB-TACE, and decreased to 38.8 (IQR 27.7–56.9), 26.1 (IQR 19.1–48.8), and 20.5 (IQR 13.1–49.7) mm at 1, 3 and 6 months later (*p* = 0.04). The ORR and DCR at 1, 3, and 6 months after DEB-TACE were 28.6% and 92.9%, 38.5% and 84.6%, 30.8% and 61.5%, respectively. The median PFS and median overall survival was 9.9 and 29.6 months, respectively.

**Conclusion:** DEB-TACE with oxaliplatin-loaded CB is suggested as a safe, effective and well-tolerated treatment for patients with unresectable or advanced lung cancer.

## Introduction

In 2022, approximately 350 deaths are projected to occur per day from lung cancer, the leading cause of cancer death in the United States (Siegel et al., 2022). Early stage lung cancer should be treated by radical resection, and simultaneous radiotherapy and chemotherapy is recommended for advanced lung cancer. Platinum-based chemotherapy, as a promising treatment, is widely used in non-small cell lung cancer, and oxaliplatin shows an excellent inhibitive effect on cancers (Ibrahim et al., 2004; Nobili et al., 2008). However, adverse effects, including cardiotoxicity and neurotoxicity, are observed and affect its efficacy in some patients due to high blood concentration (Bullinger et al., 2011; Gridelli et al., 2015). Conventional transarterial chemoembolization (TACE) and drug-eluting beads TACE (DEB-TACE) can theoretically decrease adverse events and increase the local concentration of anti-cancer drugs than conventional systemic chemotherapy (Bi et al., 2021b). In conventional TACE, a drug is carried by lipiodol and is used for arterial embolization, with no standardization of the choice of drug or embolization endpoint (Renzulli et al., 2021).

DEB-TACE, a novel drug delivery system, is characterized by arterial embolization of microspheres loaded with anti-tumor drugs and can induce tumor necrosis by embolization of the tumor-feeding arteries (Bi et al., 2021a). CalliSpheres beads (CB) has been applied for loading and releasing oxaliplatin in an *in vitro* study (Han et al., 2019), and used for the treatment of unresectable or recurrent hepatocellular carcinoma (Bi et al., 2022). Besides, DEB-TACE with gemcitabine-loaded or pirarubicin-loaded CB is feasible and well-tolerated for treatment of non-small cell lung cancer (Bie et al., 2019; Bi et al., 2021c). However, very few studies report the safety and efficacy of oxaliplatin-loaded CB in the treatment of unresectable and advanced lung cancer. This study aims to evaluate the safety and efficacy of DEB-TACE with oxaliplatin-loaded CB for treating unresectable or advanced lung cancer.

## Patients and methods

### Study design

It is a retrospective observational study and ethical approval is waived by the ethics committee of the First Affiliated Hospital of Zhengzhou University. Written informed consents were obtained from all patients. This current study included 20 patients, 11 men and 9 women (mean age 62.3 ± 14.8 years, range 33–85 years), with primary unresectable or advanced lung cancer and received DEB-TACE with oxaliplatin-loaded CB from January 2019 to December 2021.

### Patient characteristics

As shown in Table 1, 12 patients (60.0%) had adenocarcinoma and 4 patients (20.0%) had squamous cell carcinoma. There were 13 patients (65.0%) with central lung cancer and peripheral lung cancers were shown in 7 patients. Multiple tumors and single tumor were presented in 16 and 4 patients, respectively. Seven patients (35.0%) received radiotherapy and/or chemotherapy and 6 patients (30.0%) underwent targeted therapy before DEB-TACE, including bevacizumab (*n* = 2), gefitinib (*n* = 2), anlotinib (*n* = 1) and ectinib (*n* = 1).

**TABLE 1:** Patient characteristics at admission.

Variables	Data
Male, n (%)	11 (55.0%)
Mean age, years	62.3 ± 14.8
Subtype, n (%)	
Squamous cell carcinoma	4 (20.0%)
Adenocarcinoma	12 (60.0%)
Others	4 (20.0%)
Symptom duration, months	3.0 (1.0, 5.0)
Tumor length, mm	48.0 (35.2, 61.0)
Radiotherapy or chemotherapy	7 (35.0%)
Targeted therapy	7 (35.0%)
No therapy	10 (50.0%)
Single tumor/Multiple tumors	4 (20.0%)/16 (80.0%)
Central/peripheral location, n (%)	13 (65.0%)/7 (35.0%)
Local or distant metastasis	6 (30.0%)/9 (45.0%)

### Indications and exclusion criteria

The indications for DEB-TACE: age 18–85 years; pathological diagnosis of primary unresectable or advanced lung cancer; no chance or intolerable to receive surgery owing to severe heart disease, bleeding disorders; accompanied with airway stenosis or hemoptysis. Criteria of exclusion: patients with other life-threatening diseases such as heart failure or liver failure; white blood cell count <3.0 × 10^9^/L; platelets count <40.0 × 10^9^/L; breastfeeding or pregnant.

## Data collection

**FIGURE 1:**
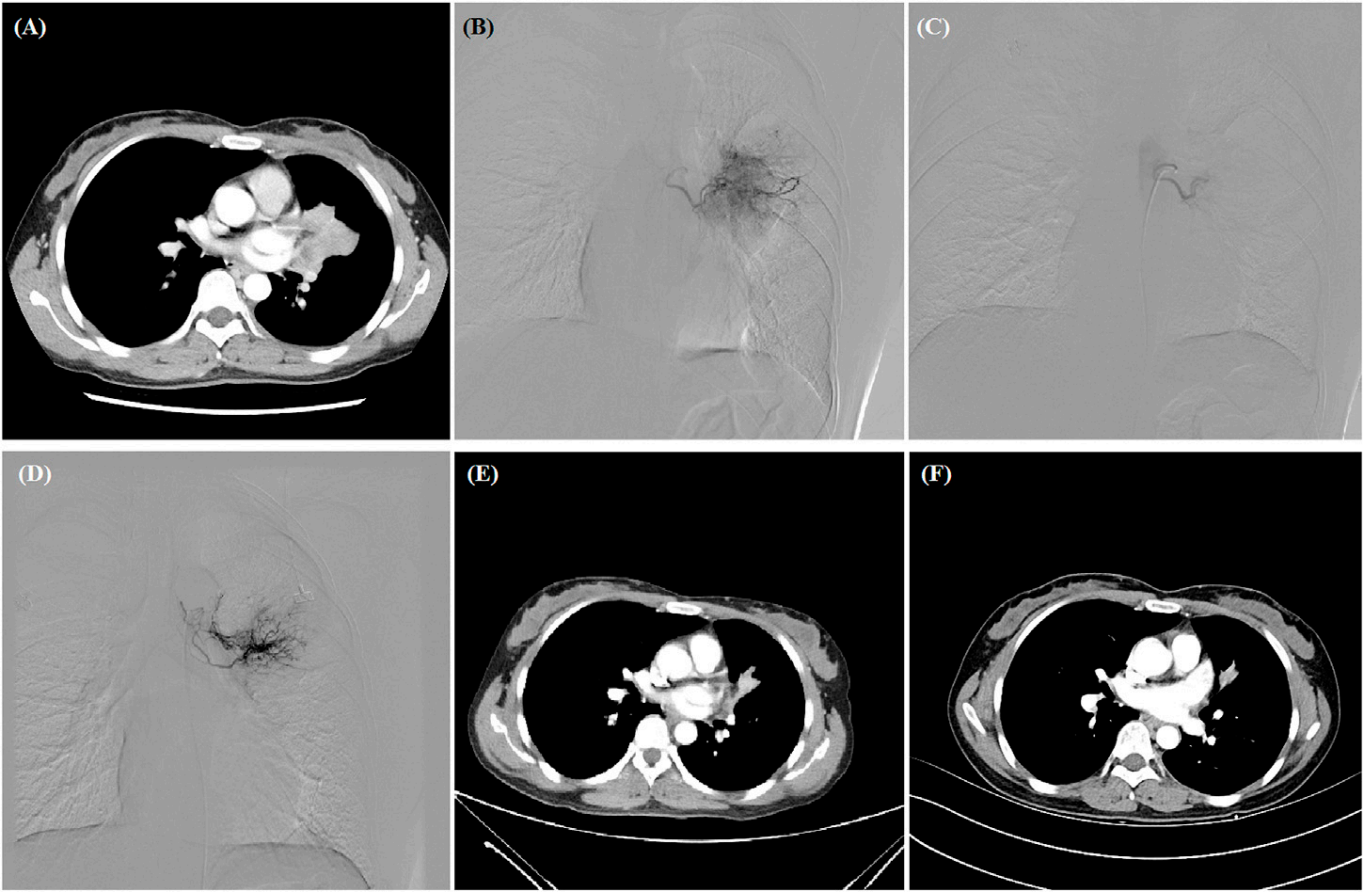
A 38-year woman with advanced adenocarcinoma treated by DEB-TACE. **(A)** CT examination revealed central malignant tumor of left lung. **(B,C)** The left bronchial artery catheterized and the blood supply artery was embolized by oxaliplatin-loaded CB. **(D)** The second DEB-TACE was performed after 2 months. **(E,F)** The left lung tumor was found to shrink at 1- and 3-month follow-up, and this woman is still alive after 36.3 months.

**FIGURE 2:**
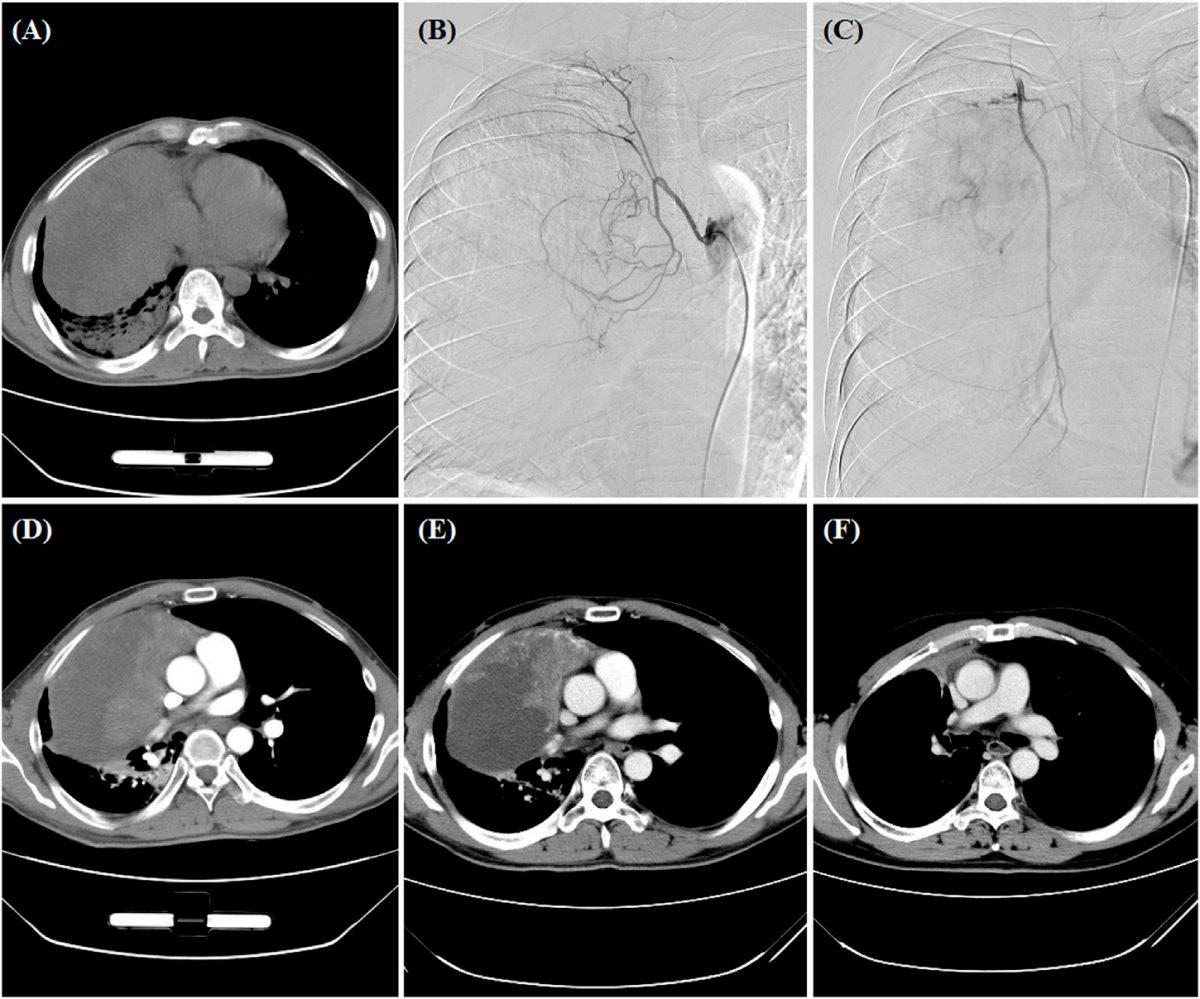
A 34-year man treated by DEB-TACE. **(A)** CT pre-procedure examination revealed a large tumor of the right lung. **(B,C)** The right bronchial artery and the right intercostal artery were the blood supply artery of the tumor. **(D,E)** The lung tumor was found to shrink at 1- and 3-month follow-up. **(F)** Surgical resection was performed after 3.6 months.

All patients underwent contrast-enhanced computed tomography (CT) examination before DEB-TACE. All patients’ baseline data were collected, including demographic data, clinical features, illness history, DEB-TACE procedure, white blood cell count, recording of chest CT, and so on (Figure 1A; Figure 2A). The tumor characteristics, such as length, location and metastasis, were shown in Table 1.

### Drug-eluting beads transarterial chemoembolization procedures

All procedures were performed under fluoroscopic guidance similar to previous report (Bi et al., 2021c). After local anesthesia, 5F-Cobra catheter (Terumo, Japan) was introduced to bronchial artery, digital subtraction angiography was performed to look for the tumor-feeding arteries and tumor staining (Figures 1B–D; Figure 2B,C). A 2.6-F microcatheter (Asahi, Japan) was inserted for super-selective catheterization. Paclitaxel (100–300 mg) or docetaxel (20–40 mg) was initially infused before embolization. Oxaliplatin (100 mg) was loaded by CB (Jiangsu Hengrui Medicine Co. Ltd., Jiangsu, China) for 30 min, and then CB was slowly injected into the tumor-feeding arteries. Polyvinyl alcohol particles (Merit, American) or gelatinum sponge particles were used if one ampoule of CB is insufficient.

### Endpoints and definitions

At about 1-, 3- and 6-month follow-up, tumor response was assessed by chest CT according to Response Evaluation Criteria in Solid Tumors version 1.1 (Eisenhauer et al., 2009). Overall survival and objective response rate (ORR) were the primary endpoints, disease control rate (DCR) and progression-free survival (PFS) were the secondary endpoints. ORR was defined as the sum of complete response and partial response, and DCR as the sum of complete response, partial response and stable disease. Adverse events were assessed according to the Common Terminology Criteria for Adverse Events (CTCAE) (version 4.0) (NCI, 2021).

### Follow-up

One patient was lost to follow-up, and the remained 19 patients were followed up by chest contrast-enhanced CT after about 1, 3, and 6 months, and then every 1–2 months after DEB-TACE (Figure 1E,F; Figures 2D–F). Patients were followed up by phone calls with the last follow-up date on 24 January 2022.

### Statistical analyses

Data of normal distribution are expressed as the mean ± standard deviation, and other data are expressed as the median (Interquartile range (IQR)) or count (%). One-way ANOVA test was used to analyze the tumor diameter change. A *p*-value <0.05 was considered statistically significant. Overall survival and PFS were calculated using the Kaplan-Meier method (Prism 5.0, GraphPad Software, Inc., SanDiego, CA).

## Results

### Drug-eluting beads transarterial chemoembolization treatments

A total of 33 sessions of DEB-TACE were administered to 20 patients, with a mean of 1.7 ± 1.0 sessions. Eight patients received a second session of DEB-TACE, with an interval of 1–3 months. A total of 55 arteries were emoblized by CB, including 40 bronchial arteries, 13 intercostal arteries, one suprarenal artery and one inferior phrenical artery. Microsphere, gelatinum sponge and polyvinyl alcohol particles were used in 3, 3, and 4 patients as supplementary embolization agents. As shown in Table 2, the median inpatient duration was 11.0 months (Rang 8.0–18.0 months) and the mean cost of hospitalizations was (6.3 ± 2.3) × 10^4^¥. Besides, one patient underwent inferior vena cava filter placement due to deep vein thrombosis. One patient received ^125^I seeds plantation for lung cancer, and airway stent implantation were performed in one patient with severe airway stricture caused by tumor invasion.

**TABLE 2:** Clinical data on DEB-TACE.

Variables	Data
Inpatient duration, months	11.0 (8.0, 18.0)
DEB-TACE sessions	1.7 ± 1.0
Total hospitalization cost, ×104 ¥	6.3 ± 2.3
Complications, n (%)	8 (40.0%)
Nausea, vomiting	2 (10.0%)
Chest pain	4 (20.0%)
Chest numbness	1 (5.0%)
Fever	1 (5.0%)
Cough	2 (10.0%)
Tumor diameter, mm	—
Before DEB-TACE	49.0 (IQR 37.8, 66.8)
After 1 month	38.8 (IQR 27.7, 56.9)
After 3 months	26.1 (IQR 19.1, 48.8)
After 6 months	20.5 (IQR 13.1, 49.7)

### Safety assessment

All DEB-TACE procedures were successfully performed, without catheter-related adverse events, nontarget embolization, or treatment-related deaths. The technical success rate of DEB-TACE was 100%. Four patients (20.0%) were complained of chest pain of grade 1, two patients showed nausea or vomiting of grade 1; those patients were well controlled within 1 week.

### Efficacy and follow up

**FIGURE 3:**
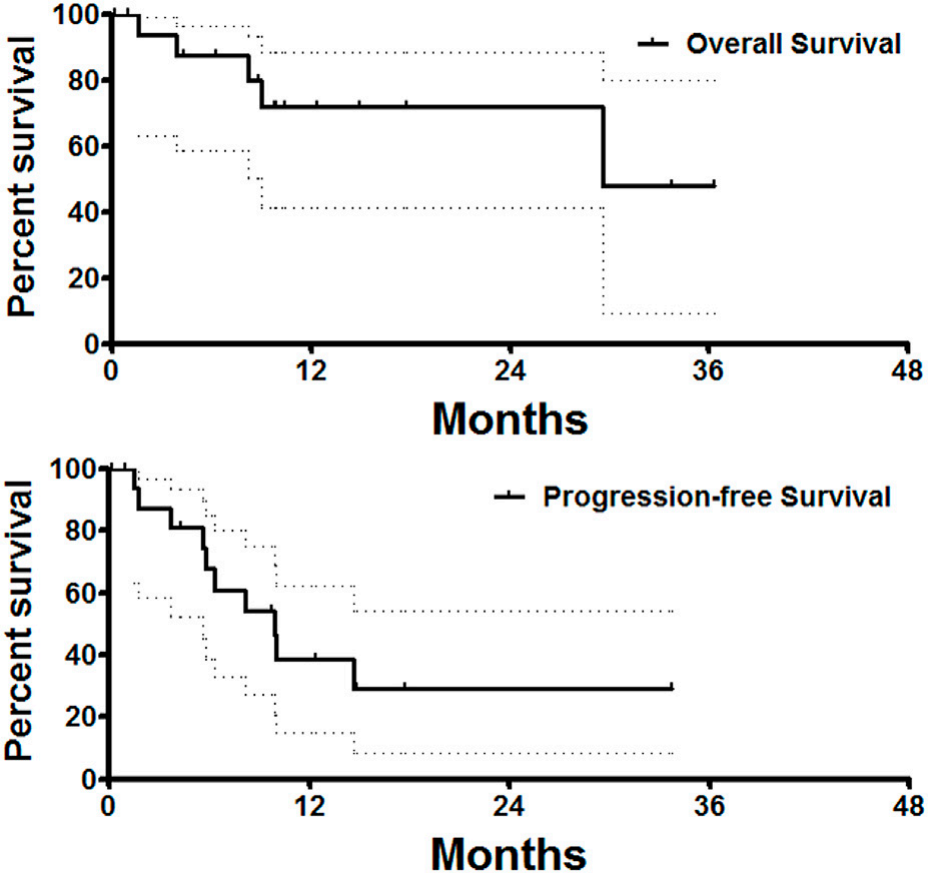
Survival follow-up. The median PFS and median overall survival was 9.9 and 29.6 months, respectively.

As the primary endpoint, the ORRs and DCRs at 1, 3, and 6 months after DEB-TACE are shown in Table 3. ORR and DCR at 1, 3, and 6 months after DEB-TACE were 28.6% and 92.9%, 38.5% and 84.6%, 30.8% and 61.5%, respectively. The median tumor diameter was 49.0 (IQR 37.8–66.8) mm before DEB-TACE, and decreased to 38.8 (IQR 27.7–56.9), 26.1 (IQR 19.1–48.8) and 20.5 (IQR 13.1–49.7) mm at 1, 3 and 6 months later (*p* = 0.04). One patient was lost to follow up (5%). The median PFS and median overall survival were 9.9 and 29.6 months, respectively (Figure 3).

**TABLE 3:** Local tumor response.

Response	1 month	3 months	6 months
Complete response	0 (0.0%)	0 (0.0%)	1 (7.7%)
Partial response	4 (28.6%)	5 (38.5%)	4 (30.8%)
Stable disease	9 (64.3%)	6 (46.2%)	4 (30.8%)
Progressive disease	1 (7.1%)	2 (15.4%)	4 (30.8%)
ORR	4 (28.6%)	5 (38.5%)	4 (30.8%)
DCR	13 (92.9%)	11 (84.6%)	8 (61.5%)

DCR, disease control rate; ORR, objective response rate.

## Discussion

Lung cancer is the leading cause of cancer death worldwide, and simultaneous radiotherapy and chemotherapy is the first choice for advanced lung cancer. Platinum-based chemotherapy, as a promising treatment, is widely used in non-small cell lung cancer, and cisplatin-based chemotherapy may improve the quality of life and increase survival (Franciosi et al., 2003). However, cisplatin has limited clinical application due to its adverse events, including cumulative neurotoxicity, nephrotoxicity, and emetogenesis (Wang and Lippard, 2005). Thus, a less toxic platinum analog, such as oxaliplatin, has been synthesized and used (Ibrahim et al., 2004; Nobili et al., 2008), which shows more significant activity but less gastrointestinal and nephrological toxicity (Monnet et al., 1998). Unfortunately, oxaliplatin is also associated with several side effects, such as cardiotoxicity and neurotoxicity, even though superior to other platinum compounds (Bullinger et al., 2011; Gridelli et al., 2015). Besides, conventional chemotherapy with high doses of drug may cause unexpected adverse event at sites other than those associated with the tumors (Jain et al., 2010).

DEB-TACE can release slowly the loaded chemotherapeutic drugs into the tumor tissue and block the blood supply of tumors. CB is the first drug-eluting beads developed in China, and have been used in many kinds of malignant tumors (Bi et al., 2021c; Bi et al., 2021d). Shang et al. (2020) reported that bronchial arterial chemoembolization with adriamycin loading CB is effective in patients with stage II-IV lung cancer. Bie et al. (2019) reported that DEB-TACE with gemcitabine-loaded CB is feasible and well-tolerated in 6 patients with non-small cell lung cancer. Although CB has been applied for loading and releasing oxaliplatin in an *in vitro* study (Han et al., 2019), the safety and efficacy of oxaliplatin loading CB have not been studied in patients with unresectable and advanced lung cancer.

It’s reported that TACE with superabsorbent polymer microspheres can alleviate symptoms and decrease tumor volume in patients with refractory lung cancer (Kennoki et al., 2015b; a), and pulmonary metastases (Seki et al., 2013). In the current study, the ORR and DCR at 1, 3, and 6 months after DEB-TACE were 28.6% and 92.9%, 38.5% and 92.9%, 38.5% and 61.5%, respectively. Our data indicated that DEB-TACE with oxaliplatin loading CB was associated with good short-term disease control rate.

For patients with metastatic lung cancer, the median overall survival was 11.3 months with chemotherapy alone (Paz-Ares et al., 2018). In our study, the median PFS and overall survival after DEB-TACE were 9.9 and 29.6 months, respectively. Our data seems better than that of DEB-TACE performed with gemcitabine-loaded CB, in which, the median PFS was 8.0 months and the median overall survival was 16.5 months (Bie et al., 2019).

For patients with larger tumor and more previous treatments, a poorer therapeutic response to DEB-TACE may be observed, and other interventional options may be able to further improve the prognosis, such as thermal ablation, transarterial infusion, targeted therapy and radioactive ^125^I seeds implantation. In this study, one patient was administered ^125^I seeds after progressive disease. Additionally, it’s also important to treat life-threatening complications caused by lung cancer, such as severe airway or esophageal stricture. In this study, airway stent implantation was performed in one patient with severe airway stricture.

As a retrospective observational study, there were some limitations. This study was conducted in a single center with no control group and the sample size was small. Studies with larger sample size and control group are wanted in the future. Previous treatment may have an interaction with DEB-TACE treatment, but the patients number was too small to analyze. Besides, only 8 patients received a second session of DEB-TACE, which may be insufficient to achieve a satisfactory efficacy.

In conclusion, DEB-TACE with oxaliplatin-loaded CB is suggested as a safe, effective and well-tolerated treatment for patients with unresectable or advanced lung cancer.

## Data Availability

The raw data supporting the conclusion of this article will be made available by the authors, without undue reservation.

## Abbreviations

DEB-TACE, drug-eluting beads transarterial chemoembolization; CB, CalliSpheres beads; ORR, objective response rate; DCR, disease control rate; PFS, progression-free survival; IQR, Interquartile range; TACE, transarterial chemoembolization.
